# The Knowledge Map of Sport and Exercise Psychology: An Integrative Perspective

**DOI:** 10.3389/fpsyg.2021.661824

**Published:** 2021-06-16

**Authors:** Alexander T. Latinjak, Antonis Hatzigeorgiadis

**Affiliations:** ^1^School of Social Sciences and Humanities, University of Suffolk, Ipswich, United Kingdom; ^2^Escola Universitària de la Salut i de l’Esport (EUSES), Universitat de Girona, Salt, Spain; ^3^Department of Physiological Education and Sport Science, University of Thessaly, Trikala, Greece

**Keywords:** athlete, mental skill, metascience, performance, physical activity, theory

## Abstract

The present work contains a personal perspective on what sport and exercise psychology (SEP) is today. It is a global synthesis of research about psychological aspects related to the context and practice of sport and exercise. The intended impact was to positively influence teaching SEP to students, to promote interdisciplinary research and practice, and to assist the development of SEP as an applied science by helping experts develop a more holistic view of the field. Over 650 theoretical and review articles about psychological concepts in connection to sport and exercise were read in the process of creating a conceptual model that reflects the essence of SEP and leads to a conceptualization of SEP based on research topics. The result was a knowledge map of SEP made up of four main research clusters: biopsychological descriptors, external variables, psychological skills, and applied SEP practice. In terms of interdisciplinarity, the present perspective on SEP suggests that sport and exercise can be used as a research paradigm or natural laboratory to study psychological aspects relevant to various scientific fields, and that sport and exercise can be used as a therapeutic framework in response to challenges that researchers and practitioners in these fields are typically addressing.

## Introduction

Sports and exercise psychology (SEP) has grown rapidly, much like some cities during the industrial revolution. New neighborhoods have emerged one after the other, and the familiar face of the old towns has dissolved into something equally bewildering and exciting. Yesterday’s SEP provides a rich legacy (see Vealey, 2006), but the development of tomorrow’s SEP could benefit from an urban map that shows what has been done and what can and needs to be done. Although the European Federation for Sport and Exercise Psychology (FEPSAC) celebrated in 2019 already its 50th anniversary, as did the International Society for Sport Psychology (ISSP) in 2015 (for an historic overview see, Elbe et al., 2019), sport psychology is only the 47th of 56 subdivisions of the American Psychological Association (APA). Nevertheless, SEP has matured, into a rapidly expanding field of psychology (Raab, 2017) spreading across many other scientific disciplines and psychological subdisciplines. SEP reached already from high performance, to education, to counseling, to organizations, to feminism, to health, to aging, to medicine, to neuroscience… As a result, there has been enough development to recognize research trends and interests to draw a map of the research landscape in SEP and provide an answer to what SEP is today.

### Aims and Scope

The aim of the present work was to present an integrative personal perspective of research on psychological aspects related to the context and practice of sport and exercise, published in SEP journals as well as other journals in psychology, sports sciences, and other scientific fields. The intended impact of this work was to positively influence teaching SEP to students, promoting interdisciplinary research and practice, and assisting the development of SEP as an applied science by helping experts develop a more holistic view of the field. SEP was initially introduced as a subject in psychology, sports science, and education in bachelor’s and postgraduate courses, later as an independent postgraduate qualification in master’s courses, but also in professionalization courses for coaches in various sports, while bachelor’s degrees in SEP were recently developed. As lecturers, a pedagogical tool in the form of a schematic model of SEP topics could help present SEP as a scientific area and introduce and contextualize module contents. In addition, when students have to decide which modules to take or to which topics they want to write their dissertation, a schematic model of SEP topics could also help them to make more informed decisions about their academic development.

Psychologists from diverse psychological disciplines have wondered if research in SEP could influence the development of their own area (Furley, 2019). In fact, the relationship between SEP and other disciplines of psychology has been intense and beneficial in a reciprocal way. On the one hand, SEP has resorted to other disciplines of psychology by applying important theories, for instance, from cultural (Ryba, 2017), clinical (Moore and Bonagura, 2017), educational (Holt et al., 2017), evolutionary (Balish et al., 2013), ecological (Araújo et al., 2019), and health (Biddle and Fuchs, 2009) psychology to the context of sports and exercise. On the other hand, SEP research can inform the research development in other areas of psychology (e.g., Shoenfelt, 2016). An integrative perspective of SEP research could facilitate the cross-pollination of the SEP literature across different psychological and other sciences to stimulate integrative theories and interdisciplinary research and practice.

For researchers who already study psychological aspects related to the context and practice of sport and exercise, an integrative perspective of the SEP research landscape can be a useful tool to discuss certain topics or results in the light of the broader literature. Given the rapid growth of SEP, especially early career researchers may find it difficult to consider the complex nomological network of concepts in which the variables of their study are embedded. SEP experts are facing an insurmountable number of publications on a variety of topics that leads many researchers to retreat into niche areas of expertise (Neimeyer et al., 2012) or insular camps (Bevan, 1991). The present work could assist researchers and practitioners to link their areas of expertise to other topics and discover new growth areas that respond to new societal challenges.

In terms of originality, apart from some introductory book chapters (e.g., Eklund and Crocker, 2019), only few global syntheses of research in SEP have been published since the beginnings of this scientific field (e.g., Browne and Mahoney, 1984). Regarding contemporary syntheses, for example, two bibliometric-review studies were conducted, on 1140 and 2276 articles, respectively (Lindahl et al., 2015; Németh et al., 2016). In both reviews, motivation, coping with anxiety and stress, physical activity behavior change, and methodological issues were identified as the most recurrent topics in SEP. Despite the undeniable contribution of these bibliometric-review studies, they identified popular research trends rather than summarizing what SEP is today (see also, Biddle, 1997; Weiss and Gill, 2005; Rhodes and Nasuti, 2011). The aim of the present work was to present a perspective of the SEP research landscape in which the key concepts found in theoretical articles and reviews are synthesized regardless of their popularity, in order to coherently reflect the scope of the SEP research in a thematic model. Furthermore, such a synthesis should also reveal the fundamental attributes required for a conceptualization of todays’ SEP based on the breadth of research topics.

## The Knowledge Map of Sport and Exercise Psychology

The perspective presented here is the result of a process similar to the three-stage model of the creative process in science by Scheffer et al. (2017): First, different inputs are collected; then these inputs are combined to novelty; finally, the result is crafted into an innovative product. In the present case, studying literature contributed to the end product along with a range of less conventional activities, including informal discussions with very different groups of people, long walks and daydreaming, and sketching models in different artistic ways. These activities might be met with skepticism in psychology, but some researchers believe they should be viewed as part of the scientific method (Scheffer et al., 2015). In addition, the view adopted in the present project accepts “a world of multiple, constructed, and thought-dependent realities” (i.e., ontological relativism; Sparkes and Smith, 2009, p. 493). Hence, it was assumed that there is no true way of explaining what SEP is. Instead, there are many idiosyncratic perspectives that can serve individuals to explain SEP to themselves and others.

### The Development of the Knowledge Map

The project began with making time for thinking and informal discussions about, and extensive reading of, 662 mostly theoretical papers and reviews that were published in SEP journals as well as in journals from psychology, sports science, and other areas (e.g., medicine or sociology). Different mental models were created and tested against each other, while reading, mindwandering, and discussing with SEP experts, other psychologists as well as coaches and athletes about what SEP is about today.

**FIGURE 1:**
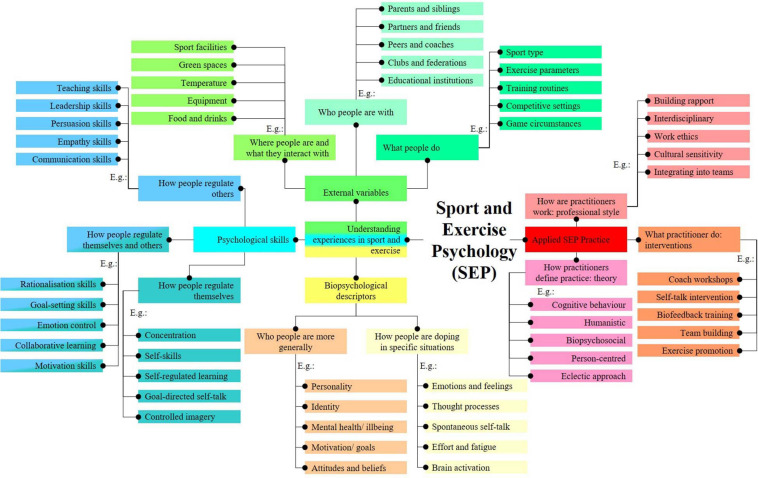
A conceptual map of sport and exercise psychology research topics.

During the development of the current perspective on SEP, the conceptual map in Figure 1 was created. SEP was placed in the center of the map, and initially a key distinction was made between two distinct but closely related goals of SEP: understanding experiences related to wellbeing and performance in sport and exercise (Biddle, 1997) and improving those experiences through *applied SEP practice* (Portenga et al., 2017). The model was further developed by recognizing that experiences related to wellbeing and performance in sport and exercise consist of internal and *external variables* (Massey et al., 2013). In addition, internal variables include both the *descriptive attributes and processes* of people and their *psychological skills* (Dohme et al., 2017). Based on the previous decisions, an outline of the knowledge map of SEP was drawn up in which SEP research concepts can be summarized in four clusters: (a) biopsychological descriptors, (b) external variables, (c) psychological skills, and (d) applied SEP practice.

With regard to the four basic clusters, topics could be identified that further organize SEP concepts. Biopsychological descriptors help answer the questions of who people are (e.g., with personality traits) and how they are in certain situations (e.g., with emotion concepts). External variables help answer the questions of where people are and what they interact with (e.g., description of sports facilities and equipment), who they are with (e.g., description of social networks), and what they do (e.g., description of task constraints). Psychological skills include concepts that refer to what people do to regulate themselves (e.g., through relaxation skills), what people do to regulate others (e.g., through modeling skills), and what people do to regulate both themselves and others (e.g., through emotion control skills). Finally, applied SEP practice research contains concepts that explain how practitioners define practice (e.g., through humanistic approaches), how practitioners work (e.g., in interdisciplinary teams), and what practitioners do (e.g., quiet eye training). In Figure 1, a selection of five examples was added to each sub-category to illustrate where a particular SEP concept might fit.

**FIGURE 2:**
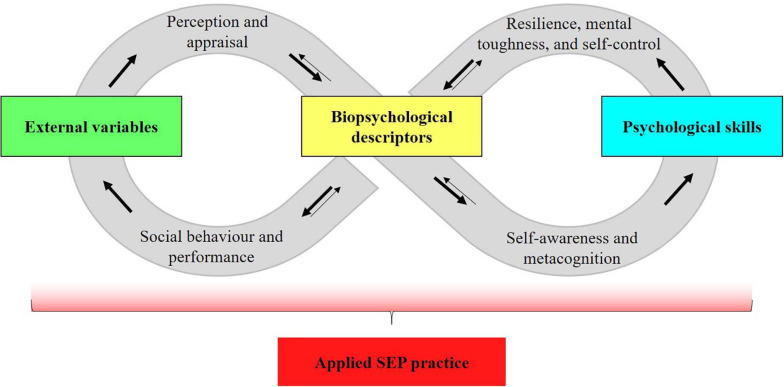
The knowledge map of sport and exercise psychology.

After elaboration, the conceptual map (Figure 1) was presented to SEP experts in conferences and informal meetings, to applied practitioners, physical education teachers and coaches in workshops, and to students in bachelor and master’s degrees. The aim was to further develop mental representations of the four main clusters and their connections to form a SEP model. The final Knowledge Map of SEP (Figure 2) was created through reading to prepare visual and written materials (e.g., this article and the figures in it), presenting the conceptual map (e.g., in conferences), and receiving positive feedback and constructive criticism (e.g., from SEP experts). Biopsychological descriptors are at the center of the knowledge map. The perception and appraisal of external variables shapes biopsychological descriptors, and biopsychological descriptors influence external variables through behavior and performance. This circular relationship can be modulated when self-awareness and metacognition trigger the use of psychological skills, the quality of this process being summarized in concepts such as resilience, mental toughness, or self-control. Finally, applied SEP practice can influence all other three clusters. The following sections detail the knowledge map by describing each major cluster and the relationships between them.

### Biopsychological Descriptors

Biopsychological descriptors are at the center of the present knowledge map because they describe how people are at certain moments and who people are in general. The cluster includes physical (e.g., height), physiological (e.g., oxygen consumption), and psychological variables (e.g., vigor) with highly variable temporal stability [i.e., processes (e.g., sadness) and attributes (e.g., personality)], which are inevitable building blocks for the experience of life. These concepts describe individuals or collectives in certain time frames, ranging from milliseconds (e.g., an adrenaline boost) to decades (e.g., a groups’ social identity). The list of concepts within this cluster is long and heterogeneous. In order to outline the conceptual landscape in SEP, it was important to find further subdivision criteria to summarize the list of biopsychological descriptors. Three partly overlapping criteria can help gain an overview of this cluster.

First, in this cluster fit physical, physiological, and psychological variables (also, anthropometric, physiological, and psychological characteristics; Baker et al., 2020), although these categories are of course not always mutually exclusive. In SEP, researchers have studied physical variables that describe people from a structural perspective within the sport and exercise context, including anatomical aspects in the brain and the rest of the body, for example, linked to an injury (Bogardus et al., 2019) or to people’s gender (Jason et al., 2015). Further, researchers have also included physiological variables that describe people on a biological functioning level, including hormonal responses (Cooke, 2013) and specific neurocognitive mechanisms (Cross et al., 2014). Although many experiences in sport and exercise could potentially be explained in terms of their physical and physiological underpinnings, SEP researchers still rely heavily on more functional psychological variables that describe people (Cross et al., 2014). For instance, concepts such body image (Srismith et al., 2020), motivation (Clancy et al., 2017), emotion (Friesen et al., 2013), beliefs (Jordana et al., 2020), and attitudes (e.g., toward doping; Morente-Sánchez and Zabala, 2013) are necessary to make sense of the uncountable interactions on a physical and physiological level in the human body.

The second criterion to organize biopsychological descriptors is related to their temporal stability. These categories are gradual and not categorically separated. Relatively stable aspects of people in or related to the context of sport and exercise are trait-like attributes and include for example age (Webdale et al., 2019) and habits (Hagger, 2019), as well as aspects related to mental health (Gucciardi et al., 2017). Rapidly changing aspects of people are state-like processes, and include, for example, conflict (Wachsmuth et al., 2017), effort (Pageaux, 2016), and attention (Moran, 2012).

Lastly, biopsychological descriptors characterize individuals and groups, the latter ranging in numbers from two individuals to broad societies. Emotions, for instance, are individual psychological processes, though they are influenced by others or similar to those that others experience at that moment (Campo et al., 2019). Motivational climate, on the other hand, is clearly a collective psychological attribute (Harwood et al., 2015), because it is not located within a single person. Overall, the cluster biopsychological descriptors includes the wide diversity of individual and collective physical, physiological, and psychological processes and attributes that describe people in and beyond the sport and exercise context.

#### Section Summary

Regarding biopsychological descriptors, it is apparent that the central focus of SEP is not limited to explaining who people are in sport and exercise contexts. SEP is equally concerned with understanding who people are from a holistic perspective (Stambulova and Wylleman, 2019) that transcends the boundaries of the world of sport and exercise. With relevance to interdisciplinary research, it should be noted that biopsychological descriptors studied in SEP are very similar to those in other areas of psychology and that the sport and exercise context may offer a special opportunity to study human nature. Many of the cultural layers present in other everyday activities often disappear in sport and exercise to reveal more primary, more rudimentary aspects of human behavior (Furley, 2019). As to future SEP research, one challenge is to uncover increasingly more biological foundations of popular psychological concepts. SEP research has already been working intensively on the measurement of psychological processes and attributes (Lindahl et al., 2015; Németh et al., 2016). In this context, understanding the biological bases of psychological concepts will become increasingly important to better study and measure these processes and attributes, and in particular the relationship between them.

### External Variables

According to SEP research, it is often the sport and exercise context that largely determines people’s psychological experiences. Increasingly popular cultural (Ryba, 2017) and socioecological approaches (e.g., Zhang and Solmon, 2013) have placed emphasis on external variables and their impact on biopsychological descriptors. Given the variety of external variables that affect people in the context of sport and exercise, including aspects as diverse as altitude of a geographic location or consequences of task failure, subcategories were needed to outline this cluster. Inspired by the categories proposed by other authors in broader literature reviews (e.g., Zhang and Solmon, 2013; Sarkar and Fletcher, 2014; Laborde et al., 2018; Bogardus et al., 2019; Webdale et al., 2019), external variables were grouped into three areas: physical environment, social and organizational influences, and task characteristics and competitive factors.

In SEP, the physical environment has been studied mainly as an antecedent or determinant of diverse biopsychological descriptors. Zhang and Solmon (2013) argued that self-determined motivation to practice physical activity is influenced by variables such as availability and convenience of physical activity facilities, safety of physical activity settings, or physical activity equipment. Laborde et al. (2018) included aromas, lights, sounds, temperature, outdoor environment, and altitude as examples of physical-environmental variables that affect cardiac vagal activity. Furthermore, Paulus et al. (2009) provided evidence that extreme cold or high altitude might be other aspects of athletes’ physical environment that can have profound effects on cognitive and behavioral processes.

Social and organizational variables used in SEP can be arranged according to the Socio-Ecological Model (e.g., Balish et al., 2014; Bogardus et al., 2019), distinguishing between the interpersonal, organizational, community, and policy level. On the interpersonal level, coaches, parents, and peers, for example, play a significant role in shaping youth sport experiences (Sheridan et al., 2014). On the organizational level, Roberts et al. (2019) identified variables such as power imbalance, conformity to dominant values, organizational tolerance, or deficient internal communication as factors related to non-accidental violence in sport. On the community level, relationships among organizations, institutions, and social networks or standards in a defined area are variables affecting health behavior (Zhang and Solmon, 2013). Finally, on the policy level, variables such as economic incentives, gender equity policy, and health and safety requirements were related to youth sport attrition (Balish et al., 2014).

Task characteristics and competitive factors include demands of a physical activity, such as whether or not task success is dependent on strength, speed, agility, flexibility, technical ability, as well as objectives and rules of a sport (Webdale et al., 2019). More specifically, Ometto et al. (2018) listed number of players involved, field dimensions and locations of play, number of goal-scoring targets used, and rules, among others, as variables that shape individual and collective tactical behaviors. Regarding more competitive factors, Sarkar and Fletcher (2014), for instance, identified match importance, score status, and momentum as variables related to stress in athletes. Finally, in research, diverse experimental conditions, such as pressure simulation tasks (Kent et al., 2018) or the Vienna Test System (Ong, 2015), are also part of task characteristics and competitive factors.

For external variables, a concept to enter this cluster should include objective and observable events rather than subjective perceptions and interpretations. The actual behavior of coaches would therefore be part of external variables, while the perception of their coach’s support by athletes would not be part of this cluster. Concepts that allude to how external variables are perceived and appraised by people fit in the cluster placed on the link between external variables and biopsychological descriptors (see next subsection). The importance of this detail in reality is best illustrated by the frequent lack of associations between observations and self-reports by athletes on coach behaviors (Smith et al., 2016).

#### Connecting Biopsychological Descriptors and External Variables

Some concepts that did not fit clearly into either biopsychological descriptors or external variables helped explaining the link between the two clusters. In the knowledge map, a bi-directional path connects biopsychological descriptors and external variables, and the concepts that describe these paths are necessarily, to some extent, both biopsychological descriptors and external variables. SEP research has studied a continuous circle in which external variables (e.g., a repetitive task) influence how and who people are (e.g., distracted), which in turn has an impact on external variables (e.g., coach’s negative reactions).

Some of the dominant theories in SEP explain parts of the interaction between external variables and biopsychological descriptors. For example, ecological dynamics to decision making assumes that behavior emerges from the performer-environment system and that both performer and environment continuously shape one another (Araújo et al., 2019). Self-determination theory suggests that the motivation to engage in physical activity is shaped by the physical and social environment in which physical activity takes place (Ng et al., 2012). Finally, flow theory describes the psychological process that involves intense focus and absorption in a particular activity, in which athletes are in harmony with their task environment without the need for effortful controlled mental processes (i.e., use of psychological skills; Swann et al., 2018). Overall, it can be assumed that the dynamic relationship between external variables and biopsychological descriptors has been one of the major research interests of SEP.

##### External variables → biopsychological descriptors

Some specific concepts refer to the influence of external variables on biopsychological descriptors which has been a central SEP research area (Zhang and Solmon, 2013; Ryba, 2017; Bogardus et al., 2019). Perception and appraisal are two concepts on this auxiliary cluster in the knowledge map. Both perception and appraisal are biopsychological descriptors that, among other things, link external variables (e.g., explicit task demands) with other biopsychological descriptors (e.g., neurologic activations in the limbic system).

In SEP research, perception had a higher physiological character in relation to basic external variables (e.g., thermal perception, Stevens et al., 2018; or visual perception, Renshaw et al., 2019) and a greater psychological character in relation to more complex external variables (e.g., perceived autonomy support, Gillison et al., 2019; or perceived safety of sport facilities, Hanlon et al., 2019). Appraisal variables inform about whether external variables are interpreted as supportive or demanding (Arnold and Fletcher, 2012), or, alternatively, as challenging or threatening (Meijen et al., 2020). Although the knowledge map primarily indicates that perception and appraisal mediate the effects of external variables on biopsychological descriptors, in reality at least the relationship between biopsychological descriptors (e.g., emotions) and perception and appraisal (e.g., challenge appraisal) is bidirectional.

##### Biopsychological descriptors → external variables

Some concepts that are inseparable from biopsychological descriptors are equally defined by their impact on external variables. To schematize SEP research, these concepts can be divided into social behavior and performance. Social behavior was defined here as verbal and non-verbal behavior that is perceptible to and has an impact on others. This list includes, among others, emotion expression (Friesen et al., 2013), communication (Beauchamp et al., 2005), leadership behavior (Cotterill and Fransen, 2016), doping (Ntoumanis et al., 2014), gambling (Derevensky et al., 2019), prosocial or anti-social behaviors (Kavussanu and Al-Yaaribi, 2019), and sport activism (Cooper et al., 2019; Valiente, 2019). Each concept in this list is part of the dynamic relationship of biopsychological descriptors, and necessarily affects external variables. For example, sports activism helps raise others’ awareness of social injustices in a broader social context (e.g., Colin Kaepernick), and doping can affect the popular image of an athlete, sport, or competition (e.g., Lance Armstrong, cycling, Tour de France).

Performance is probably one of the most central variables not only in SEP but in some of the psychological sciences and most of the sport sciences (Glazier, 2017). In most studies, performance is used as a generic concept that is defined by the way in which it is operationalized or measured (e.g., time to completion in a cycling time trial or the ISU Judging System used to judge the figure skating disciplines). Participation in physical activity and, conversely, sedentarism are two basic performance variables (Strain et al., 2019). Relatively stable changes in performance over time indicate skill acquisition (Kumari et al., 2019) or motor skill learning (Chen et al., 2018). In addition, situation-specific performance concepts include clutch performance (Swann et al., 2018) and choking (e.g., Carson and Collins, 2016), which refer to superior and inferior performance under pressure, respectively. Performance (e.g., winning a triathlon), like social behavior, is determined by biopsychological descriptors (e.g., effort and positive attitude) and materializes through changes in external variables (e.g., time on a clock, prize money, followers in social media, and world ranking). Once more, the representation in the knowledge map is simplistic, and at least the link from of biopsychological descriptors (e.g., empathy) to social behavior (e.g., prosocial-behavior) and performance (e.g., cooperation attempts) is bidirectional.

#### Section Summary

The discussions about external variables and their relation to biopsychological descriptors showed that SEP research has focused intensively on the ongoing cycle in which external variables affect individuals and collectives, and conversely, individuals and collectives influence external variables, especially through social behavior and performance. The fact that the external variables in SEP research, including typical sports and exercise tasks, influence the biopsychological descriptors that are also critical to other psychological disciplines suggests that sport and exercise can be useful as an alternative experimental paradigm. Thus, sports and exercise can be a natural laboratory for the study of psychology constructs (e.g., Sarkar and Fletcher, 2014).

Regarding future avenues for SEP research, beyond performance, scholars need to explore contemporary ways in which athletes influence their environment. For example, it is time to seriously consider how athletes influence their environment by becoming political actors (Valiente, 2019). Some athletes are challenging global societal issues and injustices such as climate change, gender inequalities, racism, sexual discrimination, or social exclusion of minorities (e.g., Cooper et al., 2019), and their actions merit researchers’ attention.

### Psychological Skills

Popular concepts in SEP research describe people’s resources to control and adjust some of their biopsychological descriptors. These resources have often been called psychological skills (also mental skills; e.g., Bányai et al., 2019). Psychological skills have long fascinated SEP researchers (Singer, 1976), possibly because they are related to the power of self-control and because, like technique, tactics, and physical condition, they can be trained (Vealey, 1988). Despite its popularity, the concept of psychological skills has fewer clear boundaries to biopsychological descriptors compared to the boundaries between those processes and attributes and external variables. In fact, biopsychological descriptors as well as psychological skills are part of people’s inner world, while external variables are part of the outer world. Therefore, the process of placing specific concepts in the cluster implicitly required a working definition of psychological skills and the description of their relationship to biopsychological descriptors.

In this work, psychological skills were conceptualized as learned intentional mental processes that control biopsychological descriptors (e.g., emotions), either to regulate those descriptors (e.g., control emotions to cope with anxiety) or to indirectly influence related descriptors (e.g., control emotions to improve attention). This conceptualization was based in parts on works like Vealey (1988), Dohme et al. (2017), and Portenga et al. (2017). To some extent, the use of psychological skills could be described as doing what one always does, but in a different way or at a different time than one would automatically or spontaneously do. In SEP, some authors have already differentiated between specific spontaneous and controlled processes (e.g., spontaneous and goal-directed self-talk; Latinjak et al., 2019). Notwithstanding the usefulness of the distinction between biopsychological descriptors and psychological skills for the purposes of the present project, caution should be exercised in distinguishing between uncontrolled and controlled processes in real life. For example, while in research the distinction between spontaneous and goal-directed (i.e., intuitive and rational) self-talk is useful, in reality the former may have some controlled rational components and the latter may be biased by spontaneous emotional aspects. Hence, although the idea of dual processes, one uncontrolled and the other controlled, may appeal seductive, the differences between biopsychological descriptors and psychological skills are in reality to be understood as a proportion difference instead of a categorical difference (Furley et al., 2015; Melnikoff and Bargh, 2018).

Specific concepts in the cluster of psychological skills differ in complexity. For example, aspects of executive functioning such as working memory, inhibitory responses, cognitive flexibility, willpower, planning, and attention control (Audiffren and André, 2015) are relatively concrete skills. However, other skills are far more complex. SEP includes research on more complex intrapersonal psychological skills including those related to emotion-control (Friesen et al., 2013), self-motivation (Chuckravanen et al., 2019), or imagery (Toth et al., 2020). In SEP more complex interpersonal psychological skills including those related to leadership (Cotterill and Fransen, 2016), persuasion (Jackson et al., 2020), or cooperative learning (Casey and Quennerstedt, 2020) have also been studied.

#### Connecting Psychological Skills and Biopsychological Descriptors

Across the SEP literature, there appears to be broad consensus that the extent to which psychological skills (e.g., leadership skills) effectively adapt some of the biopsychological descriptors (e.g., team goals) to various external variables (e.g., the score line) is critical to explaining sport performance and exercise behavior (e.g., Norris et al., 2017; Barcia et al., 2019; Liew et al., 2019). The use of psychological skills mediates the circular relationship between external variables and biopsychological descriptors. For example, coach criticism perceived by athletes as a threat can trigger muscle tension, which leads to performance decline and further criticism, unless athletes become aware of their threat appraisal or muscle tension and use psychological skills to regulate one or both to enhance performance.

Ideally, experiences like clutch performance automatically come from experiences, like flow states (Swann et al., 2018), in which the individual is in harmony with their environment. The multiaction plan model identifies this as Type I performance, characterized by automated control (Bortoli et al., 2012). If necessary, however, conscious self-regulation can lead to positive performance (i.e., Type II performance characterized by deliberate control) in two ways. Deliberate control can help create harmony (e.g., flow states) and it can effortfully prevent or counteract the negative effects of non-harmonious relationships with the environment on biopsychological descriptors. In the former case, for example, self-acceptance skills (Moore, 2009) can be important in creating flow. In the latter case, continuous efforts at self-regulation, even if they are effective at first, lead after some time to states of ego depletion (Ong, 2015). Hence, some researchers have promoted skills such as self-acceptance, which create harmony over skills used with change-oriented intentions (e.g., Birrer and Morgan, 2010).

Given the importance of psychological skills for the self-regulation of biopsychological descriptors, it is not surprising that very popular concepts can be found in both directions between the two clusters. All of these concepts have in common that they are to a certain extent defined by uncontrolled and controlled components of psychological experiences.

##### Biopsychological descriptors→psychological skills

Some concepts used in SEP are, to a certain degree, a descriptor of how people are and who people are, and a trainable ability related to self-regulation. Self-awareness (Mareš, 2019) and metacognition (MacIntyre et al., 2014) are two such concepts, that have an automatic component, like biopsychological descriptors do, but which are also part of self-regulatory processes, similar to psychological skills. Metacognition, for instance, includes *metacognitive experiences* that are more automatic feelings and judgments, and *metacognitive skills* that relate to more intentional planning, monitoring, and reviewing of task execution (for more detail see, Brick et al., 2016). These concepts at the interface between biopsychological descriptors and psychological skills (e.g., injury awareness; Chen et al., 2019) seem to be important in explaining how certain biopsychological descriptors (e.g., pain) trigger the use of psychological skills (e.g., exercise intensity control). Yet, to some degree at least the relationship between biopsychological descriptors, and self-awareness and metacognition is bidirectional.

##### Psychological skills → biopsychological descriptors

Other concepts at the interface between biopsychological descriptors and psychological skills describe the effectiveness of skills to control descriptors. Popular terms used to describe the quality of self-regulation were mental toughness (Gucciardi et al., 2017), resilience (Sarkar and Fletcher, 2014), and self-control (Englert et al., 2020). Mental toughness may be best to exemplify the mixture of descriptive processes and attributes and learned skills that compose these concepts. According to Liew et al. (2019), mental toughness is determined by inherited attributes (e.g., self-efficacy, positivity, or mental self-concept) and by learned skills (e.g., attention control or handling pressure). Furthermore, resilience refers to the positive responses to setbacks, obstacles, and failures that are essential to athletic success (Galli and Gonzalez, 2015). Self-control refers to the self’s capacity to exert control over thoughts, emotions, and behaviors (Baumeister et al., 2007). Research in SEP has linked the study of psychological skills also to failures in the process of self-regulation inserting the idea of ego depletion into the literature (e.g., Ong, 2015).

#### Section Summary

The discussions about psychological skills and their connections with biopsychological descriptors revealed that large parts of SEP research conceptualize individuals and collectives as inherently autonomous actors. As such, people play an active role in the regulation of their own biopsychological descriptors through the interaction of metacognition and psychological skills and take responsibility for their performance and their societal impact. Sport and exercise are a natural environment in which recurring psychological challenges stimulate the use and development of psychological skills. For example, young athletes need to learn to deal with fatigue, anxiety, defeat, boring routines, conflicts, inferiority, and deselection, as these are regular psychological challenges in sports and exercise (Sarkar and Fletcher, 2014). Successful individual or collective coping with these challenges strengthens the psychological skills young athletes can transfer into other areas of life (Kendellen and Camiré, 2019). Overall, SEP has provided a wealth of evidence to consider sport and exercise as potentially socializing contexts and activities (e.g., Ronkainen et al., 2020).

There is a need for further research into the delicate balance between (a) creating psychological challenges (e.g., task failure) that are essential for the development of psychological skills (e.g., self-learning skills) and (b) protecting particularly younger athletes from negative sports and exercise experiences (e.g., burnout). When psychological challenges become insurmountable, they can lead to mental illness, including burnout (Gustafsson et al., 2014) or eating disorders (Bar et al., 2016), instead of skill development. Sport and exercise psychologists have worked together with policy makers in protecting athletes from potentially harmful external stressors (e.g., combining anti-discrimination policy implementation with gender and sexual diversity education, Kavoura and Kokkonen, 2020).

What remains is the controversial question of whether positive changes in sports and education policies (Reeve, 2009), as well as in coaching and teacher education (Smith et al., 2016), have unintentionally restricted young people’s need for some of their psychological skills. According to muscle analogy (Baumeister et al., 2007), psychological skills may even decrease when not in use. Overprotection could therefore be another problem in developing psychological skills, as it may prevent challenges from occurring and skills from being trained. Overall, it seems necessary to balance some external variables (e.g., competitive demands) so that they are challenging enough to trigger the practice of psychological skills (e.g., anxiety-control skills) but not too challenging to avoid psychological distress (e.g., depression).

### Applied SEP Practice

Applied SEP practice has played a central role in this scientific field since the inception of SEP (Singer, 1976). It is therefore only logical that applied SEP practice in the knowledge map occupied a predominant position. Based on the SEP literature, field work of applied practitioners can be divided into strategic actions, targeted interventions, and therapeutic frameworks.

Strategic actions may be seen as the active ingredients in service delivery of sport and exercise psychologists (Tod et al., 2019). Examples of strategic actions reported in research include integrating into a team’s internal culture, displaying good listening skills, understanding the reality that clients face, and encouraging clients to feel safe to disclose their problems (Fortin-Guichard et al., 2018). Targeted interventions, which include diverse strategic actions, address specific problems in sport and exercise, such as coach behavior (e.g., coach development programs, Evans et al., 2015), endurance performance (e.g., music interventions, Karageorghis and Priest, 2012), skill acquisition (e.g., mental simulation practice, Moran et al., 2012), resilience (e.g., resilience-building programs, Galli and Gonzalez, 2015), attention and motivation (e.g., strategic self-talk interventions, Hatzigeorgiadis et al., 2011), or psychological skills (e.g., psychological skills training, Birrer and Morgan, 2010).

Lastly, therapeutic frameworks, which include a wide variety of strategic actions and sometimes even targeted interventions, partly define practitioners’ professional philosophy, that is, the beliefs and values that guide practitioners in their work (Fortin-Guichard et al., 2018; Poczwardowski, 2019). Concepts that refer to therapeutic frameworks and professional philosophy explored in SEP research include Rational-Emotive Behavior Therapy (Jordana et al., 2020), Cognitive Behavioral Therapy (Puig and Pummel, 2012), Motivational Interviewing (Akinrolie et al., 2020), and Mindfulness-Acceptance-Commitment approaches (Moore, 2009; see also, Poczwardowski et al., 2004). By examining strategic actions, targeted interventions, and therapeutic frameworks, researchers in SEP have mainly applied psychology knowledge and practice to improve sport and exercise experiences. However, the ultimate purpose of SEP is not focused exclusively on sport and exercise experiences, as the field also aims to have positive effects beyond sport and exercise for individuals, collectives, and societies.

Accordingly, the context and practice of sports and exercise also provide a therapeutic framework to promote, among others, positive youth development (Holt et al., 2017), physical health (Demetriou and Höner, 2012), mental health (Morres et al., 2019), and social interactions (Shvedko et al., 2018). Research has shown that sport and exercise practice can benefit a wide range of external variables, biopsychological descriptors, and psychological skills. For example, sport and exercise participation involves a social context and characteristic tasks that are important, in mental health, working memory, or social skills (Moreau and Conway, 2013). Sports and exercise practice can also elicit pleasant and challenging experiences, positive social interactions, and feelings of success and competence (Gayman et al., 2017). Moreover, participation in sport can enhance problem-solving and communication skills among young people (Holt et al., 2017). Overall, it is a remarkable feature that in SEP practice sports and exercise to are used to influence life development, as well as psychological knowledge and procedures are applied to enhance athletic experiences.

#### Section Summary

It is important to consider SEP not just as a psychological science in the service of sports and exercise experiences and performance. Sports and exercise can equally be used as a therapeutic framework for some of the social, development, health, and well-being challenges (e.g., Smith et al., 2019) that psychologists attempt to solve in other psychological disciplines. Furthermore, any psychological intervention, including sport and exercise practice, can be effective by targeting individual or combined aspects of biopsychological descriptors, external variables, and psychological skills.

In the future, researchers might want to differentiate between two potentially overlapping groups of interventions: (a) those based on changing external variables (e.g., working with athletes’ parents and coaches) and biopsychological descriptors (e.g., establishing goals for athletes) to benefit how people are, who they are, and their impact on external variables (e.g., emotions and emotion expression); and (b) those striving to fortify people’s psychological skills (e.g., emotion control skills) so that people better self-regulate their biopsychological descriptors (e.g., frustration) and positively influence their environment (e.g., through positive emotion expression). If future research further clarifies the differences and overlaps between these two groups of interventions, applied practitioners could make more explicit decisions as to whether they aim to improve individual and collective psychological skills (e.g., in talent development), or whether the situation requires them to be more directly responsible for positive sports and exercise experiences (e.g., for acute problems).

## Discussion

In the previous sections, research on psychological aspects related to sport and exercise was summarized in a knowledge map of SEP. The current section briefly discusses key insights gained from the synthesis of the SEP literature. This is mainly a possible answer to the question of what SEP is today. Based on relevant research topics, a conceptualization of SEP has emerged from the present work.

### The Conceptualization of SEP

In relation to SEP research topics, the present integrative perspective provides guidance to formulate a comprehensive conceptualization of the field. Essentially, SEP can be divided into two broad connected areas: one that investigates the dynamic relationship between biopsychological descriptors, external variables, and psychological skills; and another that looks at applied SEP practice that aims to improve that relationship. Regarding the first area, research has focused on both personal and interpersonal aspects of biopsychological descriptors and psychological skills. Regarding the second area, applied SEP practice is developed to meet two grand objectives: to improve athletic experiences (i.e., performance, social behavior, and general participant satisfaction); and to promote positive life development (i.e., life skills, socialization, physical and mental health, and general wellbeing, Schinke et al., 2016; even existential learning, Ronkainen et al., 2020) through strategic sport and exercise practice.

So, what would SEP be, considering the above aspects? SEP can be described as the science that (a) explores the dynamic network of biopsychological descriptors, external variables, and psychological skills that determine sport and exercise experiences, and (b) examines applied SEP practice that deals with different parts of this network in order to improve sport and exercise experiences and to promote positive life development through sport and exercise.

## Closing Remarks

This integrative perspective on the SEP research landscape was elaborated to answer what SEP is and what it is about. The knowledge map of SEP developed here provides a global synthesis of a scientific area that transcends disciplinary boundaries. The present knowledge map is the result of a multidisciplinary approach that included studies published in SEP as well as in other psychological sciences (Srismith et al., 2020), sports sciences (Hanlon et al., 2019), education (Casey and Quennerstedt, 2020), sociology (Valiente, 2019), health sciences (Gillison et al., 2019), physiology (Brick et al., 2016), and medicine (Bogardus et al., 2019), among others.

The present work presents a personal perspective of SEP. The perspective developed through reading, informal conversations, and free thought association during mindwandering. In terms of its true value, since this work presents perspectives intended to inspire new ideas and advancements related to SEP, it should be valued less for its accuracy than for its potential impact. The intended impact of this project was to positively influence the teaching of the SEP, to promote interdisciplinary research, and practice and to support the development of the scientific SEP area. Table 1 contains three suggestions for each of the above sub-goals and illustrative examples. The knowledge map can be useful to introduce SEP as a subject area, to present module content, and to support students in creating research questions. For researchers from other psychological disciplines and related scientific areas, the knowledge map shows common research interests, the potential of sport and exercise as ecologically valid experimental tasks, and sport and exercise as a therapeutic framework for interdisciplinary use. For SEP experts, the knowledge map can be used to examine psychological realities in applied practice (e.g., using #SportPsychMapping; Latinjak et al., in press), represent nomological networks of SEP concepts, and test the specificity of conceptualizations of research variables. To conclude, today SEP is not just a sub-discipline of psychology or sport sciences; rather, SEP would be a scientific interdisciplinary hub where interest in people’s psychological experiences converges with a psychologically, sociologically, educationally, biologically, economically, and even philosophically interesting context: sport and exercise.

**TABLE 1:** Suggestions of how the knowledge map can help teach sport and exercise psychology (SEP), establish interdisciplinary research, and to further develop the field.

Three suggestions for teaching SEP to students	(1) The knowledge map introduces SEP as a science that studies the network of biopsychological descriptors, external variables, and psychological skills, and develops practices to improve that network, to improve sports and exercise experiences, and to promote positive life development thought sport and exercise.	E.g., Students can learn that SEP describes athletes by attributes and processes such as personality and emotions that are influenced by external variables such as spectators and that may need to be adjusted by psychological skills such as anger control. SEP practitioners would help athletes to get the most out of sports practice or to use sports for their personal benefit, for example to acquire life skills.
	(2) Teachers can create a glossary of concepts that will appear during a SEP module and ask students to put them on the knowledge map and examine the relationships between these concepts based on previous knowledge and personal experience.	E.g., During a SEP module, students study task difficulty, effort, and concentration. With their teacher, students place *tasks* in external variables, *effort* in descriptors, and *concentration* in skills. Students discus that a lack of focus can lead to less effort, making tasks harder, or that difficult tasks that require more effort make it harder to concentrate.
	(3) Students writing their dissertation on a SEP topic can put their topic of interest on the knowledge map and examine related concepts in different clusters to find potentially innovative research questions.	E.g., A Ph.D. student is interested in sports anxiety. Guided by the knowledge map, they thought about studying its relation to the *descriptor* sports identity, the *external* sports culture, and self-acceptance *skills*.
Three suggestions for interdisciplinary research	(1) The knowledge map shows that SEP researchers and experts from other psychological disciplines and related sciences are interested in similar concepts. Understanding SEP research will contribute to the cross-pollination of findings between fields, thereby promoting the growth of interdisciplinary research.	E.g., Self-talk is studied in SEP as well as in other psychological disciplines and related sciences. While SEP experts develop self-talk interventions, developmental psychologists study the role of self-talk in internalizing self-regulatory processes, and educational researchers explore how self-talk facilitates mastery motivation. Yet, a lack of cross-referencing hampers the interdisciplinary development of the self-talk area.
	(2) SEP researchers have used sports and exercise tasks to manipulate similar variables that other experts in psychology and related sciences want to study. These tasks could have greater ecological validity than laboratory tasks and be therefore attractive as a research paradigm.	E.g., To study concentration and performance, researchers have used tasks like the sustained attention reaction task to control for example time, difficulty, and criteria for success. In sports tasks with potentially greater ecological validity, like golf puts or tennis returns, similar parameters can be controlled.
	(3) In SEP, sports and exercise programs have proven beneficial for individual and collective conditions, which experts from all psychological and related sciences aim at in research and practice.	E.g., Aerobic exercise in 45-min sessions at moderate intensity three times a week has a significantly large overall antidepressant effect in adults with a clinical diagnosis of major depression.
Three suggestions for SEP experts	(1) In order to examine individual and collective psychological realities, the knowledge map can help select a more holistic set of variables for interviews, field observations, objective measures, and questionnaires.	E.g., To examine an athletes’ psychological reality, a practitioner could consider observing external variables (e.g., parental behavior), measuring biopsychological descriptors (e.g., muscular tension), and asking about psychological skills (e.g., imagery).
	(2) To set up a study, relevant variables are placed into clusters and subclusters. Difficulty deciding between two or more clusters could indicate that variables in general or in the context of the study are not well enough defined.	E.g., In a study about the relations between instructor style, exercise motivation, and commitment, authors could notice that instructor style may be an external variable or a perception, motivation could be a contextual attribute or a situational process, and commitment could be an automatic descriptor or an effortful skill.
	(3) To systematically review one specific topic, the topic could be placed on the map next to other concepts that appear in its nomological network.	E.g., Prosocial and antisocial behavior were predicted by external variables like motivational climate and coach behavior, biopsychological descriptors like social identity and intrinsic motivation, and psychological skills like moral disengagement.

## Author Contributions

AL has prepared the first version of the knowledge map. AH and AL jointly developed the manuscript in its current form. Both authors contributed to the article and approved the submitted version.

## Conflict of Interest

The authors declare that the research was conducted in the absence of any commercial or financial relationships that could be construed as a potential conflict of interest.
