# Supplementary material for: The Knowledge Map of Sport and Exercise Psychology: An Integrative Perspective
Source: Front Psychol. 2021 Jun 16;12:661824. doi: 10.3389/fpsyg.2021.661824 (PMC8242169; doi:10.3389/fpsyg.2021.661824)
Supplement: Supplementary file 1 [file Table_1.docx]

| Supplementary Table  *A step-by-step description of the main phases and actions during the preparation of the conceptual map, knowledge map, and this manuscript* | |
| --- | --- |
| Main phases | Major steps |
| Defining the aims | - The aim: create an integrative perspective that reflects SEP research topics in a conceptual model. - The first intention with the model was to use it for teaching, specifically, to design a new BSc (Hons) in SEP and to introduce and contextualize module content. |
| Creating the conditions | - Time was reserved for reading, thinking, and mind wandering. - Opportunities were created to have informal conversations with colleagues in SEP and sports sciences, and applied SEP practitioners. - Further intentions emerged from early conversations:   - Inform researchers outside SEP, such as psychologists and sports scientists of what SEP is and what it is about.   - Offer applied practitioners an organized model of the complex multitude of concepts that are relevant to their daily work. |
| Searching the literature | - A Web of Science search was conducted in March 2019 - Search 1: *review* or *systematic* or *meta-analy** or *theor** as *title words* plus *sport psychology* or *exercise psychology* in *all fields*. - Search 2: *review* as *document type* in every SEP journal in the database ^a^. - This search resulted in 556 articles. - Another 106 articles have been added until the manuscript was submitted. |
| Inductive categorization | - A random sample of 200 articles were analyzed in random order. - Key concepts were identified from the title, objectives, and conclusions. - A first conceptual map was created that differentiated concepts about:   - Applied SEP practice and sport and exercise experiences.   - External variables, biopsychological descriptors, psychological skills. |
| Presenting the conceptual map | - Preparing and running presentations helped develop the conceptual map. - Essential input came from feedback in informal discussions.   - Mainly, attendees asked how the different clusters, such as biopsychological descriptors and psychological skills, are linked. - As a result, work began on the knowledge map, a SEP model. |
| Deductive categorization | - The key concepts from another random sample of 250 articles were categorized deductively. - Sub-categories of the main clusters were elaborated. - A number of concepts were identified that did not fit into a single cluster. - Those concepts, that connected the main clusters, were essential to develop the knowledge map. |
| Presenting the knowledge map | - Preparing and running presentations helped elaborating a coherent narrative about the knowledge map. - An important decision made after several presentations was to delete definitions and examples from the knowledge map so that the map contains less text that can distract from the model itself. - Most researchers and applied practitioners saw great potential in the simplified yet holistic perspective that the knowledge map offered. - Regarding the applied value of the knowledge map, some attending coaches and athletes showed great interest, as the map simplified the complex mental world. - A small fraction of experienced SEP researchers expressed unspecific doubts about the representation of SEP by the map. - It became clear that the description of the links between clusters was oversimplified and required further analysis. |
| In-depth reading | - The last 212 articles have been read in detail to determine how the knowledge map could sufficiently represent relationships between key concepts. - Bi-directional links between some main and auxiliary clusters have been highlighted. - Some important theories in SEP, such as the self-determination theory, were examined and compared with the knowledge map. - The difference between biopsychological descriptors and psychological skills was examined and described in more detail. |
| Preparing the manuscript | - To prepare the manuscript, final decisions were made about cluster names and the examples that would illustrate the concepts the clusters contain. - A co-author was invited who had previously made important contributions in several informal discussions. - Together the authors developed the summary sections at the end of each main SEP cluster section and the definition of SEP. - In the end, Table 1 was created to help the intended implications of this project for teaching, interdisciplinary collaboration, and SEP development to become a reality. - With regard to the applied value of the knowledge map, the map also served as the basis for an exploratory interview for the practice of sport and exercise psychology, which was developed in parallel with the present manuscript (Latinjak et al., 2021). |
| Submission and peer review | - Final format changes to the conceptual and knowledge maps were made for submission for peer review. - According to the anonymous reviews, the process through which the conceptual and knowledge map were developed needed further description. - The present table was created to give a step-by-step description of the review and synthesis for the SEP literature, and the manuscript elaboration. |
